# Managing the Root in Acute Type A Aortic Dissections: Are We Ready for a Standardized Approach?

**DOI:** 10.1055/s-0042-1757947

**Published:** 2023-02-27

**Authors:** Ana Lopez-Marco, Martin T. Yates, Benjamin Adams, Kulvinder Lall, John Yap, Carmelo Di Salvo, Rakesh Uppal, Aung Oo

**Affiliations:** 1 Department of Cardiothoracic Surgery, St. Bartholomew's Hospital, London, United Kingdom

**Keywords:** aortic dissection, aortic root, ascending aorta, reintervention

## Abstract

**Objectives** Surgical repair of Type A aortic dissection (TAAD) requires exclusion of the primary entry tear and reestablishment of flow into the distal true lumen. Provided that the majority of tears occur within the ascending aorta (AA), replacing only that segment seems a safe option; however, this strategy leaves the root susceptible to dilatation and need for reintervention. We aimed to review the outcomes of the two strategies: aortic root replacement (ARR) and isolated ascending aortic replacement.

**Methods** Retrospective analysis of prospectively collected data for all consecutive patients who underwent repair of acute TAAD at our institution from 2015 to 2020 was conducted. Patients were divided into two groups: (1) ARR and (2) isolated AA replacement as index operation for TAAD repair. Primary outcomes were mortality and need for reintervention during the follow-up.

**Results** A total of 194 patients were included in the study; 68 (35%) in the ARR group and 126 (65%) in the AA group. There were no significant differences in postoperative complications or in-hospital mortality (23%; *p* = 0.51) between groups. Seven patients (4.7%) died during follow-up and eight patients underwent aortic reinterventions, including proximal aortic segments (two patients) and distal procedures (six patients).

**Conclusion** Both aortic root and AA replacement are acceptable and safe techniques. The growth of an untouched root is slow, and reintervention in this aortic segment is infrequent compared with distal aortic segments, hence preserving the root could be an option for older patients provided that there is no primary tear within the root.

## Introduction

Type-A aortic dissection (TAAD) emergency repair aims to prevent death from aortic rupture or organ malperfusion. This can be accomplished by excising the primary tear and reestablishing blood flow into the true lumen; as the majority of primary tears are located within the ascending aorta (AA), supracoronary interposition graft replacement usually suffices. Any significant aortic regurgitation (AR) must be addressed by valve repair or replacement. Furthermore, the inspection of the aortic arch and proximal descending aorta by performing an open distal anastomosis under circulatory arrest and antegrade cerebral perfusion is advised to exclude the presence of intimal tears within the arch. 1 2 3

Current guidelines recommend a limited operation in the emergency setting to ensure patient survival. Aortic root replacement (ARR) is only mandated in cases where the primary tear is localized within the root and cannot be repaired due to interference with the coronary and/or aortic valve function, extensive dissection within the root, or preexisting root aneurysms 2 or when the dissection involves at least one sinus of Valsalva. 3

However, with this approach, hospital survivors may require additional surgery for native root dilatation or progression of AR. In recent years, there has been an increasing tendency toward a more extensive initial intervention with the replacement of all dissected segments within the proximal aorta, including the root and arch. 4 5 6 7 8 9 10

The aim of this study is to analyze early outcomes (survival at discharge and postoperative complications) and medium-term results (presence of residual dissection in proximal segments and need for aortic reintervention) in patients undergoing emergency ARR compared with AA replacement for TAAD.

## Materials and Methods

Retrospective analysis of prospectively collected data for all consecutive patients who underwent repair of acute TAAD in our institution from April 2015 to December 2020 was performed.

Two groups of interest were identified: (1) patients who underwent ARR and (2) patients who underwent AA replacement ± aortic valve replacement (AVR).

Patients with a primary and/or secondary entry tear within the arch mandating an arch replacement were excluded from this study ( *n* = 51).

Demographics, clinical presentation, extent of the aortic dissection, and location of the primary tear were analyzed.

Primary end points of the study were in-hospital mortality and immediate postoperative complications. Secondary end points were the presence of residual disease in the proximal aortic segments and the need of aortic reintervention.

Data were obtained from the local cardiac surgical database (Patient Advocate Tracking System, Dendrite Clinical Systems). The study was approved by our local ethical committee. Individual patient consent was waived due to the anonymized nature of the data.

### Statistical Methods

Continuous variables are expressed as median ± interquartile range, and comparison between groups was performed with the Mann–Whitney test. Categorical variables are expressed as percentages and compared using the Chi-square/Fisher exact test as appropriate.

Multiple logistic and Cox's regression models were used to identify the predictors of early and late mortality including all the significant variables listed in the annexed tables (cut-off at *p* < 0.05; Table 1 ). The results were expressed as odds ratios (ORs) and hazard ratios (HRs) with corresponding 95% confidence intervals (CIs).

**Table 1:** Preoperative characteristics for patients undergoing acute Type A aortic dissection repair, grouped according the surgical procedure: aortic root replacement (ARR) or ascending aorta (AA) replacement for the nonmatched and the propensity score matched groups

	ARR ( *n* = 68, 35%)		AA ( *n* = 126, 65%)			ARR matched ( *n* = 68)		AA matched ( *n* = 68)		
	*n*	%/range	*n*	%/range	*p*	*n*	%/range	*n*	%/range	*p*
Age (y) Median ± IQR	55 ± 31	20–86	63 ± 23	19–89	0.41	55 ± 31	20–83	62.5 ± 21	19–85	0.73
Female sex	19	27.9	50	39.7	0.11	19	27.9	23	33.8	0.46
Hypertension	45	66.2	101	80.2	**0.03**	45	66.2	56	82.3	**0.03**
Diabetes	3	4.4	10	7.9	0.54	3	4.4	5	7.3	0.72
Hypercholesterolemia	11	16.2	24	19	0.62	11	16.2	15	22.1	0.38
Current smoker	14	20.6	19	15.1	0.4	2	2.9	4	5.9	0.68
Ex smoker	13	19.1	19	15.1	0.4	13	19.1	9	13.2	0.63
Prior cardiac surgery	6	8.8	3	2.4	**0.02**	6	8.8	2	2.9	**0.02**
Prior CVA	2	2.9	6	4.8	0.71	2	2.9	4	5.9	0.68
COPD	2	2.9	9	7.1	0.33	2	2.9	5	7.3	0.44
Creatinine Median ± IQR	94.5 ± 36	50–235	104 ± 54	51–800	0.57	94.5 ± 39	6–235	112 ± 54	(51–800)	0.38
Connective tissue disorder	5	7.3	2	1.6	0.13	5	7.3	2	2.9	0.24
Bicuspid aortic valve	1	1.5	1	0.8	0.08	1	1.5	–		0.91
Cardiac tamponade	10	14.7	20	15.9	0.83	10	14.7	11	16.2	0.81
*Clinical malperfusion* :										
Coronary	4	5.9	6	4.8	0.11	4	5.9	2	2.9	0.07
Neurological	1	1.5	9	7.1	**0.02**	1	1.5	3	4.4	0.05
Abdominal	3	4.4	2	1.6	0.06	3	4.4	1	1.5	0.05
Limb	6	8.8	10	7.9	0.11	6	8.8	7	10.3	0.08
EuroScore II	10.2 ± 16	2–80	12.1 ± 25	2–80	0.38	12.2 ± 16	2–80	9.1 ± 9	2–53	0.38

Abbreviations: AVR, aortic valve replacement; CVA, cerebrovascular accident; COPD, chronic obstructive pulmonary disease; IQR, interquartile range.

As the two groups were significantly different with respect to their baseline characteristics, propensity score matching (with a match tolerance of 0.05) was performed (including the preoperative characteristics including in Table 1 ). The matched groups were analyzed using the methods described above.

A Kaplan–Meier survival analysis was done, comparing the curves by the log-rank statistic.

Statistical Package for the Social Sciences 27.0 was used to analyze the data.

### Surgical Technique

All cases were performed via median sternotomy. Cannulation strategies, use of deep hypothermic circulatory arrest, and strategies for cerebral perfusion varied among the operating surgeon and the years of the study. That resulted in a variety of cannulation strategies (femoral 49.5%, AA/arch 46.4%, axillary 3.1%), targeted core temperatures (mean: 21°C, range: 15–30°C), and 24 cases done with minimal/no circulatory arrest. When cerebral perfusion was used, the antegrade versus retrograde route was preferred (130 vs. 5 patients).

The institutional policy recommends ARR in the presence of a tear within the sinuses of Valsalva or at the origin of the coronary ostia or for a root diameter ≥ 50 mm. In young patients, those with connective tissue disorders and those with severely diseased aortic valves, the root is replaced at a lower diameter, according to individual criteria.

For dissected aortic root layers (but no intimal tear within the root), the repair consists of removal of thrombus from the false lumen and reconstruction of the tissues including two strips of Teflon inside and outside the aortic wall and a buttress anastomosis. We do advocate for a two-layered anastomosis (first layer with continuous polypropylene suture and the second layer with interrupted pledgeted polypropylene sutures) at both proximal and distal locations to reduce the intraoperative bleeding and the midterm complications of the suture line due to the persistence of entry tears. The use of biological glue has been slowly abandoned in our practice because of reported toxicity and associated pseudoaneurysms. 11 12

A dedicated emergency rota of seven surgeons, with specialist training in aortovascular surgery, was established in our institution in September 2020. This has resulted in the standardization of surgical techniques (advocating for more extensive repairs in the index operation and routine use of the two-layered anastomosis) and adjuncts for organ protection (distal anastomosis performed under CA, preferred antegrade cannulation routes and administration of antegrade cerebral perfusion and nasopharyngeal/core temperature dropped to 22°C).

### Follow-up Imaging

The protocol for postoperative follow-up after TAAD repair in our institution includes gated contrast-enhanced CT of the whole aorta and transthoracic echocardiography (TTE) predischarge and at 3, 6,12 months and then annually.

The presence of residual dissection in the aortic root was defined by the identification of a dissection flap within the aortic root and the presence of contrast or color Doppler signal in that lumen by CT and/or TTE.

Pseudoaneurysm was defined as a contained leak with surrounding hematoma extending from the vicinity of the aortic anastomotic line in the absence of a dissection flap.

## Results

A total of 194 patients underwent TAAD repair; 68 (35%) underwent ARR and 126 (65%) underwent AA replacement. After propensity score matching, the sample was reduced to 136 patients, 68 in each group.

### Demographics and Dissection Characteristics

Patients who received ARR were younger (55 ± 31 vs. 62.5 ± 21 years, *p* = 0.73). Cardiovascular risk factors (except hypertension), as well as comorbidities, were similar in both groups. Prior cardiac surgery was significantly more prevalent in the ARR group (8.8 vs. 2.9%, *p* = 0.02).

Patients presenting with neurological malperfusion underwent AA repair more frequently (7.1 vs. 1.5%, *p* = 0.02; Table 1 ).

The pattern of the dissection was significantly different between groups. Patients who received an ARR presented with a more extensive dissection and more community not only affecting the root (64.7 vs. 39.7%) but also the aortic arch and more distal segments ( *p* < 0.005).

Twenty-six patients with a tear outside the root underwent ARR based on other factors such as root dimension, young age, and connective tissue disease. A higher degree of AR was more frequent in those who underwent ARR compared with those who received AA ± AVR (severe AR: 32.4 vs. 8.8%, *p* = 0.01).

The size of the aortic root on presentation influenced the surgical technique, with larger roots in the ARR group ( *p* < 0.05; Table 2 ).

**Table 2:** Anatomy and extension of the acute Type A aortic dissection for the two surgical groups: aortic root replacement (ARR) or ascending aorta (AA) replacement for the nonmatched and the propensity score matched groups

	ARR ( *n* = 68, 35%)		AA ( *n* = 126, 65%)			ARR matched ( *n* = 68)		AA matched ( *n* = 68)		
	*n*	%	*n*	%	*p*	*n*	%	*n*	%	*p*
*Extension dissection* :										
Root	44	64.7	53	42.1	**0.003**	44	64.7	27	39.7	**0.008**
Arch	37	54.4	61	48.4	0.42	37	54.4	34	50	0.61
DTA	30	44.1	50	39.7	0.55	30	44.1	29	42.6	0.86
Visceral aorta	26	38.2	40	31.7	0.36	26	38.2	22	32.4	0.47
Infrarenal aorta	21	30.9	32	25.4	0.41	21	30.9	16	23.5	0.33
*Intimal tear* :										
Root	27	39.7	–		**0.001**	24	35.3	–		**0.004**
Ascending	26	38.2	69	54.7	**0.001**	7	10.3	36	94.7	**0.004**
*Intraoperative toe* :										
Moderate AR	16	23.5	20	15.9	**0.001**	16	23.5	14	20.6	**0.01**
Severe AR	22	32.4	13	10.3	**0.001**	22	32.4	6	8.8	**0.01**
Poor LV	4	5.9	5	3.9	0.56	4	5.9	–		0.05
*Size aortic root* :										
< 40 mm	15	22.1	50	39.7	**0.001**	15	22.1	30	44.1	**0.004**
40–50 mm	24	35.3	31	24.6	**0.001**	24	35.3	13	19.1	**0.004**
> 50 mm	19	27.9	7	5.6	**0.001**	19	27.9	5	7.4	**0.004**
AVR	–		22	17.5	**0.001**	–		10	14.7	**0.001**

Abbreviations: AR, aortic regurgitation; AVR, aortic valve replacement; DTA, descending thoracic aorta, LV, left ventricle.

Analysis of the preoperative CT scans at the time of the study was available in 145 patients (74.7%).

### Operative Data

AVR was performed in 17% of the cases (22 patients) who received AA replacement based on severe AR not amenable to repair by commissural resuspension or based on the presence of a degree of aortic stenosis; choice of valve prosthesis was biological ( *n* = 12, 54.5%) and mechanical ( *n* = 10, 45.5%).

For those who received ARR, the choice of prosthesis was mechanical ( *n* = 39, 57.3%), biological ( *n* = 24, 35.3%), and native valve (valve-sparing root replacement, *n* = 5, 7.3%). Open distal anastomosis with the hypothermic circulatory arrest was performed in 170 patients (87.6%) and cerebral perfusion was used in 147 patients (75%). Median temperature targeted for circulatory arrest was 22°C (15–30°C).

Salvage operation was defined as patients who experienced cardiac arrest and required cardiac massage prior to sternotomy or establishment of cardiopulmonary bypass.

The addition of ARR increased the cardiopulmonary bypass and aortic cross-clamp times (258 ± 90 vs. 199 ± 68 and 158 ± 73 vs. 102 ± 55 minutes, respectively; Table 3 ).

**Table 3:** Timing of surgery, cannulation strategies, cerebral protection strategies, and surgical times for the two surgical groups: aortic root replacement (ARR) or ascending aorta (AA) replacement for the nonmatched and the propensity score matched groups

	ARR ( *n* = 68, 35%)		AA ( *n* = 126, 65%)			ARR matched ( *n* = 68)		AA matched ( *n* = 68)		
	*n*	%/range	*n*	%/range	*p*	*n*	%/range	*n*	%/range	*p*
Emergency	65	95.6	117	92.8	0.22	65	95.6	65	95.6	0.47
Salvage	3	4.4	9	7.1	0.22	3	4.4	3	4.4	0.47
CPB (min) Median ± IQR	258 ± 90	79–580	199 ± 68	77–456	0.15	258 ± 90	79–580	198 ± 67	77–456	0.35
AXC (min) Median ± IQR	158.5 ± 73	54–394	102 ± 55	46–258	0.13	158.5 ± 73	54–394	101 ± 48	47–258	0.27
CA (min) Median ± IQR	29.5 ± 29	0–76	27.5 ± 22	0–154	0.22	29.5 ± 29	0–76	28 ± 26	0–93	0.42
DHCA	58	85.3	112	88.9	0.40	58	85.3	59	86.8	0.44
Cerebral protection	41	60.3	94	74.6	**0.01**	41	60.3	60	88.2	**0.02**
Core temperature (degrees celsius Median ± IQR)	22 ± 7	15–30	22 ± 4	15–30	0.52	22 ± 6	18–30	22 ± 16	15–30	0.40
*Arterial cannulation* :										
Ascending aorta/arch	33	48.5	57	45.2	0.47	33	48.5	30	44.1	0.35
Axillary artery	3	4.4	3	2.4	0.47	3	4.4	1	1.5	0.35
Femoral artery	32	47.1	64	50.8	0.47	32	47.1	35	51.5	0.35

Abbreviations: AXC, aortic cross-clamp; CA, circulatory arrest; CPB, cardiopulmonary bypass; DHCA, deep hypothermic circulatory arrest; IQR, interquartile range.

### Early Results

There was no significant difference in postoperative complications or in-hospital mortality (23%) between the groups ( Table 4 ).

**Table 4:** Postoperative complications, mortality and rate of aortic reintervention, and residual aortic disease for the two surgical groups: aortic root replacement (ARR) or ascending aorta (AA) replacement for the nonmatched and the propensity score matched groups

	ARR ( *n* = 68, 35%)		AA ( *n* = 126, 65%)			ARR matched ( *n* = 68)		AA matched ( *n* = 68)		
	*n*	%	*n*	%	*p*	*n*	%	*n*	%	*p*
Stroke	12	17.6	39	31	0.11	12	17.6	23	33.8	0.08
Hemofiltration	20	29.4	35	27.8	0.42	20	29.4	17	25	0.25
Tracheostomy	11	16.2	22	17.5	0.43	11	16.2	10	14.7	0.31
Hospital mortality	16	23.5	29	23	0.51	16	23.5	14	20.6	0.54
Late mortality	2	2.9	5	3.9	0.99	1	1.5	5	7.3	0.19
Reintervention	1	1.5	7	5.6	0.57	1	1.5	4	5.9	0.53
Residual disease	5	7.3	11	8.7	0.48	5	7.3	8	11.8	0.28

Causes of death were cardiac (40%), neurological (22%), multiorgan failure (15%), abdominal (6%), respiratory (4%), intraoperative exsanguination (2%), and unknown (11%).

Multivariate regression analysis identified presentation with coronary malperfusion (OR: 4.4, 95% CI: 1.2–16.2, *p* = 0.03), cardiac tamponade (OR: 2.6, 95% CI: 1.1–6.2, *p* = 0.02), and salvage operation (OR: 9.5, 95% CI: 1.9–47.7) as independent predictors of in-hospital mortality.

### Midterm Results

Follow-up was up to 5.8 years, with a median of 2.5 (0.1–5.8) years. Twenty patients (10.3%) were lost to follow-up, although survival data were 100% completed via the National Household Survey census.

Seven patients (4.7%) died during follow-up, five (3.9%) in the AA group and two (2.9%) in the ARR group ( *p* = 0.99; Table 4 ). Causes of death were neurological (28.6%), cancer (28.6%), pneumonia (14.3%), and unknown (28.6%).

**Fig. 1:**
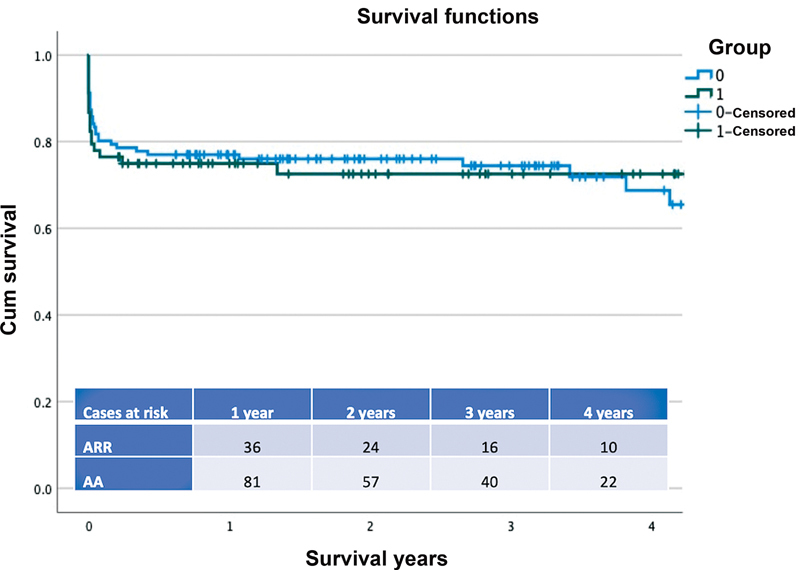
The Kaplan–Meier survival curve for patients who underwent acute Type A aortic dissection repair with ascending aorta (group 0) replacement or aortic root replacement (group 1). Log rank *p* = 0.95. The number of patients at risk in each group at the different time frames is also provided in the annexed table. AA, ascending aorta; ARR, aortic root replacement.

**Fig. 2:**
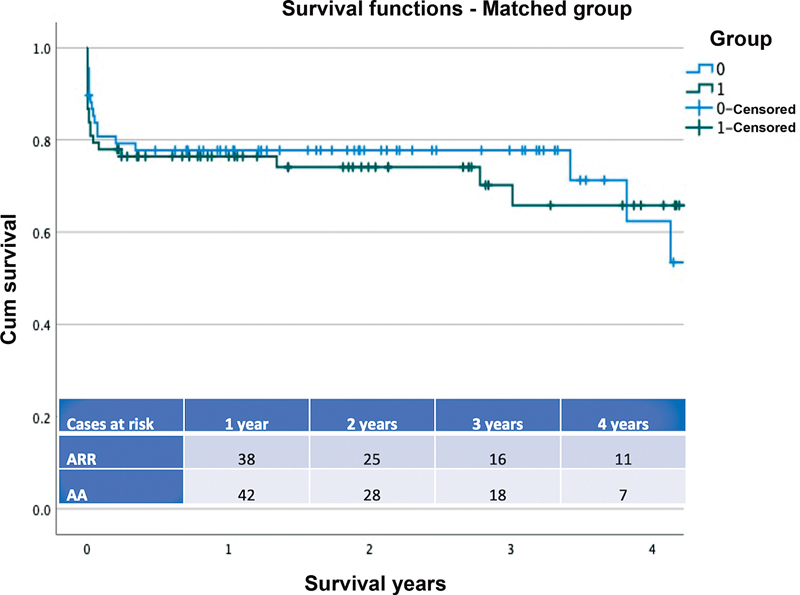
The Kaplan–Meier survival curve for patients who underwent acute Type A aortic dissection repair with ascending aorta (group 0) replacement or aortic root replacement (group 1) in the propensity score matched population. Log rank *p* = 0.72. The number of patients at risk in each group at the different time frames is also provided in the annexed table. AA, ascending aorta; ARR, aortic root replacement.

The Kaplan–Meier analysis estimated survival of 74.9% (95% CI: 3.47–4.75) in the ARR group and 76.5% in the AA group (95% CI: 3.75–4.67) at 1 year, and 72.5% in the ARR group and 73.1% in the AA group at 3 years, long rank *p* = 0.95 ( Figs. 1 – 2 ).

Predictors of late mortality identified by Cox regression were preoperative and postoperative stroke (HR: 4.7, 95% CI: 1.3–17.6, *p* = 0.02), postoperative hemofiltration (HR: 0.2, 95% CI: 0.05–0.1, *p* = 0.001), and tracheostomy (HR: 3.5, 95% CI: 1.4–8.9, *p* = 0.01).

### Aortic Reinterventions and Significant Residual Aortic Disease

A total of eight patients underwent aortic reinterventions, including proximal aortic segments (two patients developed pseudoaneurysms in the proximal anastomosis) and distal procedures (six patients requiring arch replacement with frozen elephant trunk [one patient], descending thoracic aorta replacement [three patients, 37.5%], or extent II thoracoabdominal aneurysm repair [two patients]). The mean age of this reintervention subgroup was 42.5 (29–64) years, 33.4% were females, and 22.2% had connective tissue disease. The reintervention occurred at 18.1 (3–42) months after the index procedure, which has been AA replacement in the majority of patients.

**Fig. 3:**
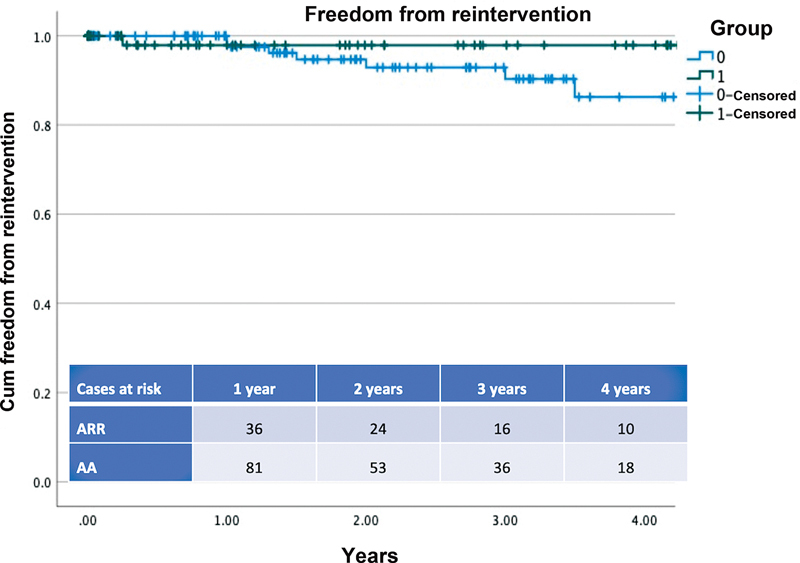
The Kaplan–Meier curve representing freedom from aortic reintervention for patients who underwent acute Type A aortic dissection repair with ascending aorta (group 0) replacement or aortic root replacement (group 1) log rank *p* = 0.24. The number of patients at risk in each group at the different time frames is also provided in the annexed table. AA, ascending aorta; ARR, aortic root replacement.

Freedom from reintervention at 1 year calculated by Kaplan–Meier analysis was 97.9 ± 2.1 years in the ARR group and 94.7 ± 2.6 years in the AA group (log rank *p* = 0.24; Fig. 3 ).

Cox's regression identified preoperative aortic root dimension as an independent factor for reoperation, with an increased risk for those >50 mm (HR: 0.01, 95% CI: 0.7–0.9, *p* = 0.01).

Significant residual disease, with an indication for intervention, was present in 16 patients, either in isolation or as a combination of two segments: 7 of them had a residual dissection flap in the root, 2 patients had residual dissection in the distal AA, 2 patients had aortic arch aneurysms (54 and 66 mm, respectively), 1 patient had a new dissection flap in the innominate artery (used for intraoperative aortic cannulation), and 5 patients had a pseudoaneurysm of the aortic anastomosis.

Those with residual dissection of the aortic root, by definition, were in the AA group. The mean age of these patients was 62.8 years (38–77), 50% were females, mean aortic root dimensions were 40.4 mm (31–54 mm), and the time since the index operation was 2.6 years (1–5). None of these patients had more than mild AR.

Complications in the distal aortic segments were not related to the index aortic procedure ( *p* = 0.42). Two of these patients died 2 to 3 years after the index procedures, with one of them having been intervened for a chronic aortic graft infection that caused further anastomotic dehiscence.

## Discussion

There is no consensus regarding the best approach for managing the aortic root in TAAD. The literature to date is contradictory—some studies show no difference, whereas others indicate higher operative mortality when performing ARR. Also, there is inconsistency as to whether preserving the aortic root is a risk factor for later reoperation. 4 5 6 7 8 9 12 13 14 15 16 17 18 19

In our series, those who underwent ARR for TAAD repair were more often younger, with connective tissue disorder or bicuspid aortic valve (BAV), with more proximal location of the intimal tear, as well as more extensive distal dissection with larger aortic root dimensions.

The addition of ARR replacement inevitably increased surgical times but did not impact the circulatory arrest times or cerebral protection strategy. Based on this, increased complications might have been expected for the ARR group, but both in-hospital mortality and postoperative complications were equivalent for the two groups.

We agree with the recommendations made by Yang et al. 15 ARR should be considered as the index procedure for TAAD in patients with an intimal tear in the root, preexisting root aneurysms (>45 mm), connective tissue disorders, BAV (when presenting with root dilatation >45 mm and/or valve disease), and/or unrepairable aortic valve pathology. Nishida et al 16 also recommended ARR in cases of extensive aortic root dissection, affecting at least two sinuses of Valsalva. They demonstrated clear association with rapid root dilatation (>3 mm/year), progressive valve incompetency, development of pseudoaneurysms, and reoperations for those treated with root repair in this context.

Several techniques to repair a dissected root have been described, 11 12 13 14 15 including the use of biological glue or inserting Teflon felt between the dissected layers prior to performing the buttress suture. We do not advocate for either, but rather ensure all the thrombus is removed and the buttressed suture is performed with two bands of Teflon to prevent residual entry tears. The blood pressure after the repair will push the dissected layers together provided that there is no residual entry tear. Preservation of a dissected aortic root always has a potential risk of recurrence: aortic root dissection, aneurysm/pseudoaneurysm formation, severe AR, and reoperation. In our study, although we did not have to reintervene in many patients for these reasons, we have cases being closely monitored due to residual root dissection, which eventually may need to be addressed.

About 30% of TAAD survivors face a reoperation during their life, especially during the first 5 years. However, it seems that the determining factor is the distal rather than the proximal aorta. 10 18 19 Reinterventions in the proximal segments are extremely infrequent and the freedom from intervention does not seem to be influenced by the aortic root treatment. 20 Freedom from root interventions has been reported at 99% after 3, 88 to 95% after 5, and 77 to 83% after 10 years. 4 5 14 17 However, other causes of surgical recurrence such as pseudoaneurysms, infective endocarditis, and AR are not necessarily excluded by performing ARR. 9

In our series, most reoperations were to treat expansion of distal segments or complications of the index procedures. We have patients under surveillance for residual root dissection, which could have been prevented either by a more aggressive approach in the index operation or more accurate proximal repair of the dissected tissues. However, it has been demonstrated that the distal aorta grows faster than the proximal aorta and that proximal dissected aortas behave similarly to aneurysmal aortas, with a dilatation rate of 0.4 mm/y. 12 Therefore, patients with residual root dissection and mild aneurysms might be safely managed with close surveillance until their aortas start dilating and/or they develop significant AR.

## Conclusion

Both ARR and AA replacement are acceptable and safe techniques for TAAD repair.

A more aggressive approach should be considered based on patient factors (age, presence of connective tissue diseases) and aortic factors (dimension of the root, location of the primary tear, and extent of the dissection).

Reintervention in proximal aortic segments is less frequent than in distal segments, hence preserving the root could be an attractive option for older patients with other comorbidities provided that there is no primary tear within the root.

Our study has the limitations of being a single-center retrospective experience and with a short follow-up period.

## References

[1] Tsai T T Nienaber C A Eagle K A Acute aortic syndromes Circulation 2005 112 24 3802 3813 16344407 10.1161/CIRCULATIONAHA.105.534198

[2] Hiratzka L F Bakris G L Beckman J A 2010 ACCF/AHA/AATS/ACR/ASA/SCA/SCAI/SIR/STS/SVM guidelines for the diagnosis and management of patients with Thoracic Aortic Disease: a report of the American College of Cardiology Foundation/American Heart Association Task Force on Practice Guidelines, American Association for Thoracic Surgery, American College of Radiology, American Stroke Association, Society of Cardiovascular Anesthesiologists, Society for Cardiovascular Angiography and Interventions, Society of Interventional Radiology, Society of Thoracic Surgeons, and Society for Vascular Medicine Circulation 2010 121 13 e266 e369 20233780 10.1161/CIR.0b013e3181d4739e

[3] ESC Committee for Practice Guidelines; The Task Force for the Diagnosis and Treatment of Aortic Diseases of the European Society of Cardiology (ESC) Erbel R Aboyans V Boileau C 2014 ESC guidelines on the diagnosis and treatment of aortic diseases: document covering acute and chronic aortic diseases of the thoracic and abdominal aorta of the adult Eur Heart J 2014 35 41 2873 2926 25173340 10.1093/eurheartj/ehu281

[4] Di Eusanio M Trimarchi S Peterson M D Root replacement surgery versus more conservative management during type A acute aortic dissection repair Ann Thorac Surg 2014 98 06 2078 2084 25282163 10.1016/j.athoracsur.2014.06.070

[5] Hysi I Juthier F Fabre O Aortic root surgery improves long-term survival after acute type A aortic dissection Int J Cardiol 2015 184 285 290 25731839 10.1016/j.ijcard.2015.02.020

[6] Leshnower B G Chen E P When and how to replace the aortic root in type A aortic dissection Ann Cardiothorac Surg 2016 5 04 377 382 27563551 10.21037/acs.2016.03.15 PMC4973127

[7] Urbanski P P Lenos A Irimie V Bougioukakis P Zacher M Diegeler A Acute aortic dissection involving the root: operative and long-term outcome after curative proximal repair Interact Cardiovasc Thorac Surg 2016 22 05 620 626 26848190 10.1093/icvts/ivw002 PMC4892147

[8] Ikeno Y Yokawa K Yamanaka K The fate of aortic root and aortic regurgitation after supracoronary ascending aortic replacement for acute type A aortic dissection J Thorac Cardiovasc Surg 2021 161 02 483 493.e1 31839222 10.1016/j.jtcvs.2019.09.183

[9] Castrovinci S Pacini D Di Marco L Surgical management of aortic root in type A acute aortic dissection: a propensity-score analysis Eur J Cardiothorac Surg 2016 50 02 223 229 26941248 10.1093/ejcts/ezw038

[10] EACTS/ESVS scientific document group Czerny M Schmidli J Adler S Current options and recommendations for the treatment of thoracic aortic pathologies involving the aortic arch: an expert consensus document of the European Association for Cardio-Thoracic surgery (EACTS) and the European Society for Vascular Surgery (ESVS) Eur J Cardiothorac Surg 2019 55 01 133 162 30312382 10.1093/ejcts/ezy313

[11] Kazui T Washiyama N Bashar A HM Role of biologic glue repair of proximal aortic dissection in the development of early and midterm redissection of the aortic root Ann Thorac Surg 2001 72 02 509 514 11515890 10.1016/s0003-4975(01)02777-1

[12] Peterss S Dumfarth J Rizzo J A Sparing the aortic root in acute aortic dissection type A: risk reduction and restored integrity of the untouched root Eur J Cardiothorac Surg 2016 50 02 232 239 26916934 10.1093/ejcts/ezw012

[13] Rylski B Beyersdorf F Blanke P Supracoronary ascending aortic replacement in patients with acute aortic dissection type A: what happens to the aortic root in the long run? J Thorac Cardiovasc Surg 2013 146 02 285 290 22841905 10.1016/j.jtcvs.2012.07.013

[14] Lai D T Miller D C Mitchell R S Acute type A aortic dissection complicated by aortic regurgitation: composite valve graft versus separate valve graft versus conservative valve repair J Thorac Cardiovasc Surg 2003 126 06 1978 1986 14688716 10.1016/s0022-5223(03)01279-0

[15] Yang B Norton E L Hobbs R Short- and long-term outcomes or aortic root repair and replacement in patient undergoing acute type A aortic dissection repair: twenty-year experience J Thorac Cardiovasc Surg 2019 157 215 236 10.1016/j.jtcvs.2018.09.129 PMC6588514 30737109

[16] Nishida H Tabata M Fukui T Takanashi S Surgical strategy and outcome for aortic root in patients undergoing repair of acute type A aortic dissection Ann Thorac Surg 2016 101 04 1464 1469 26627176 10.1016/j.athoracsur.2015.10.007

[17] Geirsson A Bavaria J E Swarr D Fate of the residual distal and proximal aorta after acute type a dissection repair using a contemporary surgical reconstruction algorithm Ann Thorac Surg 2007 84 06 1955 1964, discussion 1955–1964 18036916 10.1016/j.athoracsur.2007.07.017

[18] Estrera A L Miller C C III Villa M A Proximal reoperations after repaired acute type A aortic dissection Ann Thorac Surg 2007 83 05 1603 1608, discussion 1608–1609 17462365 10.1016/j.athoracsur.2007.01.029

[19] Concistrè G Casali G Santaniello E Reoperation after surgical correction of acute type A aortic dissection: risk factor analysis Ann Thorac Surg 2012 93 02 450 455 22206955 10.1016/j.athoracsur.2011.10.059

[20] Ellauzi H Zafar M A Wu J Fate of preserved aortic root following acute type A aortic dissection repair Semin Thorac Cardiovasc Surg2021; 2022;34 02 419 427 33979665 10.1053/j.semtcvs.2021.04.002

